# The zinc finger transcription factor PW1/PEG3 restrains murine beta cell cycling

**DOI:** 10.1007/s00125-016-3954-z

**Published:** 2016-04-29

**Authors:** Mozhdeh Sojoodi, Leslie Stradiot, Karo Tanaka, Yves Heremans, Gunter Leuckx, Vanessa Besson, Willem Staels, Mark Van de Casteele, Giovanna Marazzi, David Sassoon, Harry Heimberg, Paola Bonfanti

**Affiliations:** Diabetes Research Center, Vrije Universiteit Brussel, Laarbeeklaan 103, 1090 Brussels, Belgium; Stem Cells and Regenerative Medicine Team, Institute of Cardiology and Nutrition, Inserm UMRS-1166, University Pierre and Marie Curie (Paris VI), Paris, France; Institute of Child Health, University College London, 30 Guilford Street, WC1N 1EH London, UK; Institute of Immunity and Transplantation, University College London, London, UK

**Keywords:** Beta cell, Pancreas development, *Peg3*, Proliferation, *Pw1*

## Abstract

**Aims/hypothesis:**

*Pw1* or paternally-expressed gene 3 (*Peg3*) encodes a zinc finger transcription factor that is widely expressed during mouse embryonic development and later restricted to multiple somatic stem cell lineages in the adult. The aim of the present study was to define *Pw1* expression in the embryonic and adult pancreas and investigate its role in the beta cell cycle in *Pw1* wild-type and mutant mice.

**Methods:**

We analysed PW1 expression by immunohistochemistry in pancreas of nonpregant and pregnant mice and following injury by partial duct ligation. Its role in the beta cell cycle was studied in vivo using a novel conditional knockout mouse and in vitro by lentivirus-mediated gene knockdown.

**Results:**

We showed that PW1 is expressed in early pancreatic progenitors at E9.5 but becomes progressively restricted to fully differentiated beta cells as they become established after birth and withdraw from the cell cycle. Notably, PW1 expression declines when beta cells are induced to proliferate and loss of PW1 function activates the beta cell cycle.

**Conclusions/interpretation:**

These results indicate that PW1 is a co-regulator of the beta cell cycle and can thus be considered a novel therapeutic target in diabetes.

**Electronic supplementary material:**

The online version of this article (doi:10.1007/s00125-016-3954-z) contains peer-reviewed but unedited supplementary material, which is available to authorised users.

## Introduction

Under physiological conditions, beta cell division declines continuously after birth in both humans and rodents [1]. In response to increased metabolic demand such as obesity or pregnancy, beta cells can undergo hyperplasia and hypertrophy suggesting that their innate capacity to divide is not lost in the adult [2].

The *Pw1* (also known as *Peg3*) parentally imprinted gene is unique to placental mammals and encodes a large protein containing a Krüppel-type zinc finger [3, 4]. Chromatin immunoprecipitation sequencing analyses have shown that PW1 controls multiple genes that regulate the cell cycle and/or cellular metabolism [5–7]. Furthermore, in vitro studies have shown that PW1 mediates p53–Bax signalling in the p53 growth arrest and cell death pathway activated by DNA damage, possibly by interacting with Siah1 to induce Bax translocation to the mitochondria [8–10]. Consistent with these in vitro studies, loss of *Pw1* expression correlates with increased cell proliferation and tumour grade in gynaecological and glioma cell lines [6, 7, 11]. Taken together, these studies suggest that PW1 acts as a cell cycle inhibitor and tumour suppressor in multiple cell types. However, *Pw1* expression, while widespread during early mouse embryonic development [4], is progressively downregulated in most tissues during late embryonic and fetal development. At these stages, high levels of expression are restricted to adult somatic stem cells in several tissues, including skeletal muscle, gut, hair follicles, the central nervous system and bone marrow [12]. The ‘pan-stem cell’ pattern of *Pw1* expression in the adult prompted us to examine its expression in the pancreas during embryonic development and adulthood.

## Methods

### Mice

All animal experiments were approved by our institutional Ethical Committee for Animal Experiments and followed national guidelines and regulations. BALB/c adult and timed pregnant mice were purchased from Janvier Labs (St Berthevin, France). Male C57BL/6 mice of 8 weeks old underwent partial duct ligation (PDL) surgery, as previously described [13]. We generated a new mouse strain with a novel conditional allele, *Pw1*^+/lox^. Male *Pw1*^+/pat−^ mice (F3) were crossed with C57BL/6 female mice (Janvier Lab) to obtain age-matched paternal knockout and wild-type littermates. Four to six mice per cage were maintained under a 12 h light/dark cycle at 24°C and fed a standard diet ad libitum. *Pgk*^Cre^, *Ptf1a*^Cre^*Ngn3*^YFP^ and *Ngn3*^−/−^ strains were described previously [14–16]. These transgenic mice had mixed genetic backgrounds.

### Immunostaining and image analysis

Tissue sections and cultured cells were processed for immunostaining as previously described [17]. Sections were incubated overnight at 4°C with primary antibodies (electronic supplementary material [ESM] Table 1) diluted in 0.2% normal donkey serum containing 0.01% Triton X-100. Cyanine- or DyLight-labelled secondary antibodies were obtained from Jackson ImmunoResearch (Newmarket, UK). The total beta cell volume was analysed as previously described [17–19]. Images were captured with an inverted microscope (Nikon Instruments, Amstelveen, the Netherlands) equipped with a Hamamatsu digital camera c10600 (Olympus, Tokyo, Japan) or with a multiphoton microscope (Zeiss LSM710 NLO with a TiSa laser, NY, USA) and analysed using Smartcapture 3 (version 3.0.8) NIS AR2.30 Imaging Software (Nikon France, Champigny-sur-Marne, France) or Improvision Volocity LE (version 5.0) (PerkinElmer, Waltham, MA, USA) and ImageJ software (https://imagej.nih.gov/ij/) [20].

### Lentivirus-mediated gene knockdown

A *Pw1* targeting sequence (5′-GAGTCGCAGTCAATCGATT-3′), loop sequence (5′-TTCAAGAGA-3′) and its reverse complement were annealed and cloned into the *Bgl*II and *Hind*III sites of the pSuper.basic vector. Insert-containing clones were selected by PCR and verified by sequencing. Next, an *Eco*RI–*Sal*I fragment containing the H1 promoter and the cloned oligo duplex was subcloned into the *Bam*HI–*Cla*I sites of the pTripGFP vector and co-transfected with the pMD2g and pCMV∆8.4 plasmids into 293 T cells, as described previously [21]. Viral vectors were obtained from P. Ravassard (ICM - Institut du Cerveau et de la Moelle épinière, Hôpital Pitié-Salpêtrière, Paris, France) and 293 T cells from ATCC (LGC Standards, Molsheim Cedex, France). Viruses were collected from the culture medium, filtered and concentrated by ultracentrifugation.

### RNA analysis

Total RNA isolation, amplification and normalisation of data were done as previously described [17]. Primer and probe sequences for quantitative PCR are presented in ESM Table 2.

### Statistical analysis

Data are expressed as the means ± SEM of at least three independent experiments and analysed by unpaired two-tailed Student’s *t* test or two-way and one-way ANOVA followed by Tukey’s honest significant difference post hoc testing. A *p* value of <0.05 was considered statistically significant. Sample sizes are shown in the figure legends.

## Results

### PW1 expression in the pancreas of embryonic and adult mice

**Fig. 1:**
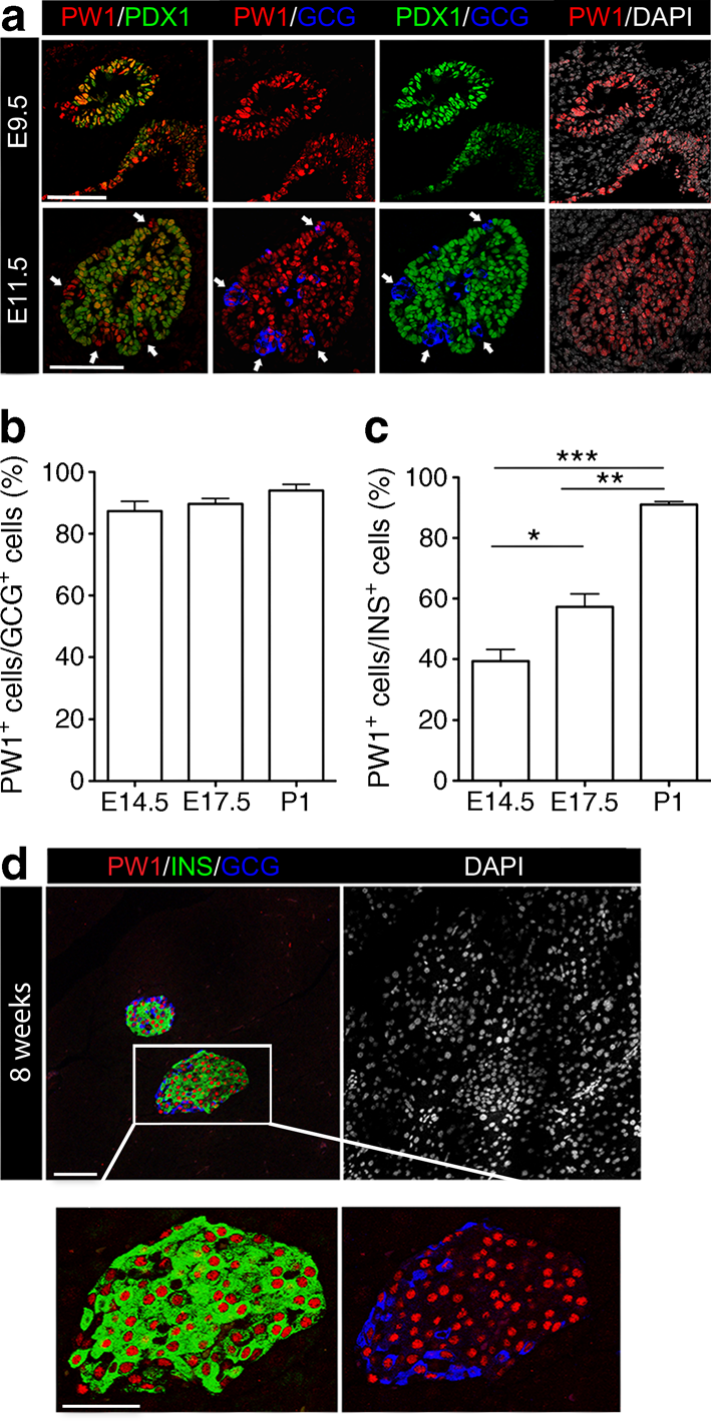
Biphasic expression pattern of PW1 during pancreatic development. (**a**) Expression of PW1 (red), PDX1 (green) and GCG (blue) in the pancreases of E9.5 and E11.5 mice. White arrows indicate GCG^+^ PW1^+^ cells. Scale bars 100 μm. (**b**, **c**) Relative amounts of GCG^+^ and INS^+^ cells that express PW1 at E14.5, E17.5 and P1 (*n* = 3, mean ± SEM, *p < 0.05, **p < 0.01, ***p < 0.001; one-way ANOVA). (**d**) Expression of PW1 (red) in the adult pancreas (INS, green; GCG, blue). Scale bars 50 μm

During the primary transition phase of the developing mouse pancreas (embryonic day [E]9.5–E11.5), PW1 is detected in the nuclei of pancreatic and duodenal homeobox 1 (PDX1)^+^ progenitor cells (Fig. 1a). Glucagon-expressing (GCG^+^) cells are generated during this period and most contain high levels of PW1 throughout gestation (Fig. 1a, b). In contrast, the number of insulin-expressing (INS^+^) cells that express PW1 progressively increases during embryonic development (Fig. 1c). During the secondary transition phase (E13.5–E15.5), the number of PW1^+^ PDX1^+^ cells decreases and most PW1 is found in cells lining and near to the exocrine ducts at sites where endocrine progenitor cells become specified (ESM Fig. 1a–c). In addition, in pancreases of *Ngn3* knockout mice that lack endocrine cells [14], PW1^+^ cells were present in both mutant and wild-type littermates at an early stage of pancreatic development (E11.5–E14.5) but absent after endocrine specification (at E17 and later) in *Ngn3* knockout mice (ESM Fig. 1d). In the adult pancreas (at 8 weeks old), PW1 was abundant in most beta cells (only 4.64 ± 0.35% of INS^+^ cells were PW1^−^) and alpha cells (Fig. 1d) but also in somatostatin^+^ delta cells and pancreatic polypeptide^+^ cells (data not shown). We conclude that PW1 is expressed in all pancreatic progenitors before endocrine specification and primarily in the endocrine cells after specification.

### PW1 restrains the beta cell cycle

**Fig. 2:**
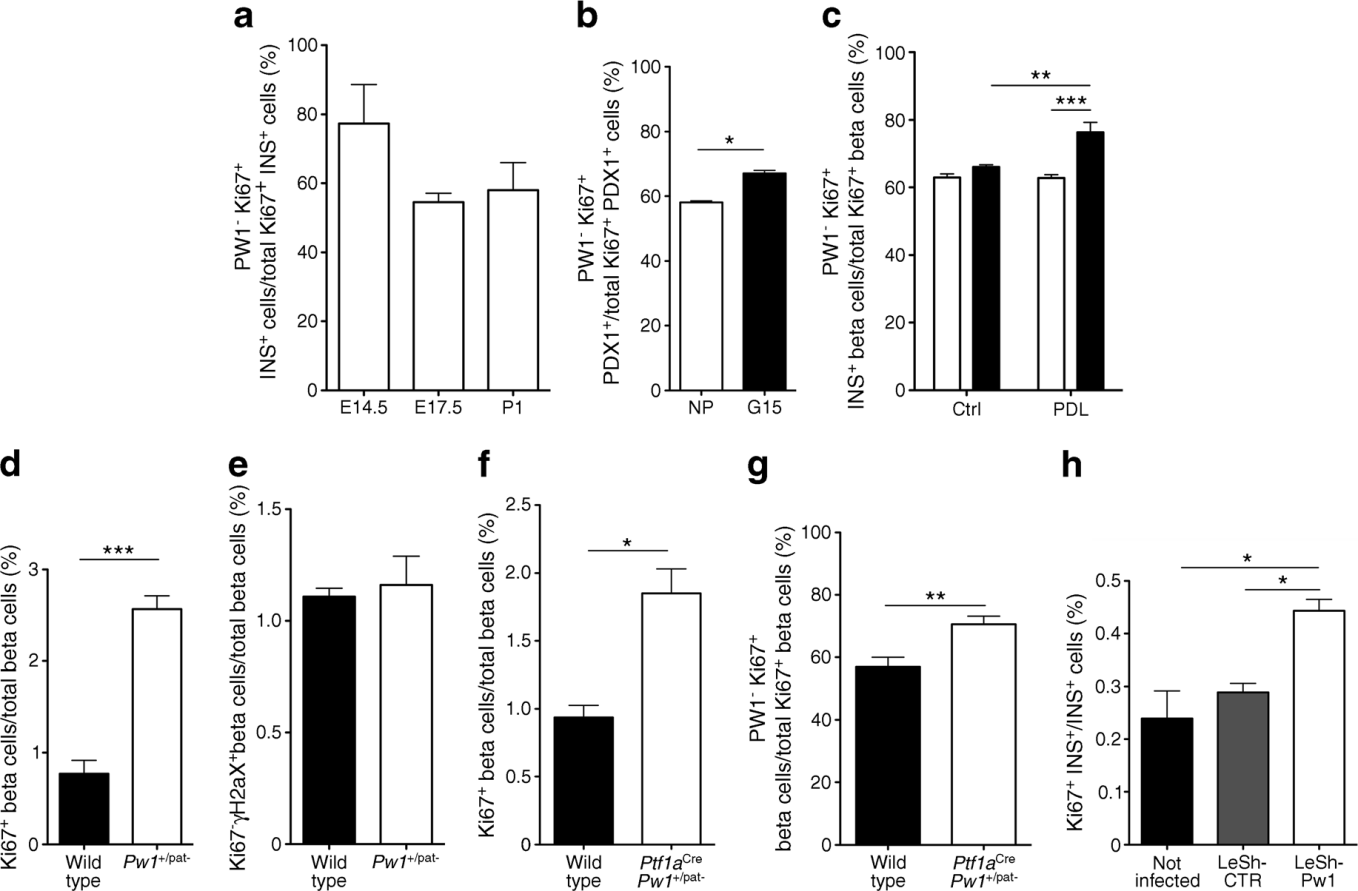
PW1 expression inversely correlates with beta cell cycling. (**a**) Percentage of cycling (Ki67^+^) PW1^−^ INS^+^ cells out of the total number of cycling PDX1^+^ cells at E14.5, E17.5 and P1. (**b**) Percentage of cycling PW1^−^ PDX1^+^ cells at gestational day 15 (G15) out of the total number of cycling PDX1^+^ cells compared with non-pregnant (NP) mice (*n* = 3, mean ± SEM, *p < 0.05; Student’s *t* test). (**c**) Percentage of cycling PW1^−^ INS^+^ beta cells out of the total number of cycling beta cells in controls (Ctrl) or the ligated (tail, white bars) and unligated (head, black bars) parts of PDL pancreases (*n* = 3, mean ± SEM, **p < 0.01, ***p < 0.001; one-way ANOVA). (**d**) Percentage of cycling beta cells out of the total number of beta cells in *Pw1* ^+/pat−^ mice (*n* = 3, mean ± SEM, ***p < 0.001; Student’s *t* test). (**e**) Percentage of γ-H2aX^+^ cells out of the total number of beta cells in wild-type and *Pw1* ^+/pat−^ mice. (**f**) Percentage of cycling beta cells out of the total number of beta cells in wild-type and *Ptf1a* ^Cre^ *Pw1* ^+/pat−^ mice (*n* = 3, mean ± SEM, *p < 0.05; Student’s *t* test). (**g**) Percentage of cycling PW1^−^ beta cells out of the total number of cycling beta cells in wild-type and *Ptf1a* ^Cre^ *Pw1* ^+/pat−^ mice (*n* = 3, mean ± SEM, **p < 0.01; Student’s *t* test). (**h**) Percentage of cycling INS^+^ cells out of the total number of INS^+^ cells in islet cells either uninfected or transduced with LeShCTR or LeShPw1 (*n* = 3, mean ± SEM, *p < 0.05; one-way ANOVA)

As PW1 is involved in growth arrest in several cell types, we investigated its presence in cycling beta cells. In the pancreases of embryonic mice at E14.5 and E17.5, as well as in newborn mice, most actively cycling beta cells do not express PW1 (Fig. 2a). In 8-week-old mice, 0.79 ± 0.17% of all INS^+^ cells were Ki67^+^; of these, 59 ± 8% were PW1^−^. The number of PW1^+^ beta cells in the pancreases of 8-week-old mice is 4% higher than in postnatal day 1 (P1) and 14% higher than in P5. In adult mice, beta cell proliferation can be activated by pregnancy [2] or in response to injury by PDL [22]. The abundance of *Pw1* mRNA was decreased in islets from mice at gestation day 15 compared with islets of non-pregnant mice, and also in islets from the ligated part of the pancreas tail after PDL vs after sham treatment (data not shown). Thus, under both conditions, PW1 levels inversely correlated with the beta cell proliferation index (Fig. 2b, c).

To determine whether PW1 plays an active role in controlling the beta cell cycle, we investigated loss of *Pw1* function in mice derived from a cross between *Pgk*^Cre^ mice and a novel conditional *Pw1* allele that we generated, *Pw1*^+/lox^. Offspring from these crosses were bred onto a C57BL/6 background for at least seven generations and the *Cre* allele was then eliminated to generate a constitutive PW1-null mutant mouse in which part of *Pw1* exon 8 and the entire coding domain of exon 9 were deleted. These *Pw1*-null mutant mice, hereafter called *Pw1*^+/pat−^, are viable but show a 20% postnatal growth reduction [23]. The pancreases of 8-week-old *Pw1*^+/pat−^ mice lacked PW1 (ESM Fig. 2a) but showed no evidence of gross anatomical abnormalities, exhibited normal cellular architecture in both endocrine and exocrine compartments, and had normal expression patterns of endocrine (PDX1, INS and GCG) and exocrine (amylase and CK19) cell markers (ESM Fig. 2b). However, we noted that the percentage of Ki67^+^ beta cells was significantly increased in *Pw1*-null mutant mice compared with their wild-type littermates (Fig. 2d). Remarkably, we did not detect a difference in the levels of DNA damage marker γ-H2aX foci between the two groups (Fig. 2e), suggesting that the higher number of cycling beta cells is not a consequence of increased beta cell death. On the other hand, the total beta cell volume was not increased in pancreas of *Pw1*^+/pat−^ mice, suggesting that the cell cycle is activated but not completed in beta cells of *Pw1*-null mutant mice.

Beta cell specificity of this phenomenon was examined in *Pw1*^+/lox^ mice crossed with *Ptf1a*^Cre^ mice, which express Cre recombinase under the control of the pancreas transcription factor 1a gene (*Ptf1a*) promoter and hereafter are called *Ptf1a*^Cre^*Pw1*^+/pat−^ mice. Notably, the *Ptf1a* promoter is activated in most, but not all, of the early pancreas progenitor (PDX1^+^) cells at E9.5 (ESM Fig. 2c, upper panels), and is essential for pancreas specification and function in both mice and humans [15]. Recombination efficiency in the adult beta cells of *Ptf1a*^Cre^*Pw1*^+/pat−^ mice was 71 ± 14% (*n* = 3). PW1 was expressed in PDX1^+^ cells and thus also in PTF1A^+^ cells from wild-type mice (ESM Fig. 2c, lower panels). The pancreases of *Ptf1a*^Cre^*Pw1*^+/pat−^ mice developed normally and showed normal architecture and cell composition (data not shown). However, there was a twofold increase in the number of Ki67^+^ PDX1^+^ or INS^+^ cells in the pancreases of 8-week-old *Ptf1a*^Cre^*Pw1*^+/pat−^ mice compared with their wild-type littermates (Fig. 2f). Further, the fraction of Ki67^+^ beta cells that did not express PW1 was significantly increased (Fig. 2g), demonstrating that actively cycling beta cells are mostly negative for PW1. As Ptf1a is not expressed in all pancreatic progenitor cells [15], *Pw1* was not deleted from the entire progenitor population, and consequently the entire beta cell population, leading to underestimation of the effect of PW1 on beta cell cycling.

To confirm a direct role for PW1 in beta cell cycling, its expression was downregulated by transduction of beta cells with recombinant lentiviruses expressing either a short hairpin (sh)RNA that specifically targets mouse *Pw1* (LeShPw1) or a control sequence (LeShCTR), together with green fluorescent protein. Infection efficiency at a multiplicity of infection of 100 was at least 65%, and the level of PW1 protein was efficiently reduced in LeShPw1- compared with LeShCTR-transduced cells (ESM Fig. 2d). After 7 days in culture, the number of Ki67^+^ LeShPw1-transduced beta cells had increased 1.5-fold compared with Ki67^+^ LeShCTR-transduced beta cells (Fig. 2h). Taken together, our data demonstrate that downregulation of PW1 triggers cell cycle initiation in beta cells.

## Discussion

The present study reports the expression pattern of PW1 in the developing, postnatal and adult pancreas both under healthy conditions and after injury. Our analyses revealed that PW1 co-localises with PDX1 in E9.5 pancreatic buds. At later stages, the expression of PW1 became progressively restricted to pro-endocrine cells, and ultimately to the islets of Langerhans after birth. PDX1, a homeodomain transcription factor necessary for normal development of the pancreas [24], is transiently downregulated during pancreatic lineage specification, and the same spatiotemporal expression pattern is seen for PW1. Following endocrine cell specification, PW1 levels increased but only in differentiated endocrine cells. This biphasic pattern of expression is also observed for other key pancreatic transcription factors, including PDX1 [25]. Previous studies have shown that PW1 is found in a wide array of bona fide adult somatic stem/progenitor cells, including those of bone marrow, skin, bone and muscle [12]. Ongoing studies in other tissues show that when PW1 function is lost, the progenitors continue to expand rapidly but fail to self-renew, ultimately resulting in a net loss of progenitor cells and a failure to maintain regenerative capacity. Therefore, the expression of PW1 in beta cells is unusual because these cells are fully differentiated and are consequently not considered to represent a stem cell compartment [26]. Nevertheless, since PDX1 is not only necessary for pancreas specification but also essential for adult beta cells [27] and since PW1 controls the cycling of other cell types [10, 28], we investigated whether PW1 affects beta cell cycling in the embryonic and postnatal pancreas. During pancreatic development, a gradual increase of the number of PW1^+^ INS^+^ cells coincides with a decrease in the beta cell proliferation rate. The number of actively cycling beta cells is decreased in adult compared with neonatal mice, which contain fewer PW1^+^ cells. Previous reports revealed that PW1 acts as a p53 mediator through direct interaction with the Siah1 protein to control the cell death pathway and regulate cell cycle arrest via interaction with TNF receptor-associated factor 2 (TRAF2) and Bcl2-associated X (BAX) [10]. Although beta cells respond to stress, we found that the major role of PW1 is to restrain the endocrine cell cycle, a finding that has not previously been reported for primary adult differentiated cells. In addition, conditions that stimulate beta cell proliferation such as pregnancy and pancreatic injury correlated with a decrease in PW1 expression, further supporting a role for PW1 as a negative regulator of the beta cell cycle. Consistent with this proposed role, we observed that loss of PW1 function in mutant mice resulted in significant activation of the beta cell cycle postnatally compared with their wild-type littermates. However, increased beta cell cycling did not result in an increased total beta cell volume, suggesting that PW1 might prime beta cells for proliferation by allowing G1 phase entry, while additional constraints control the continuation and completion of the cell cycle, thus preventing an increase in cell number. Moreover, a shRNA-mediated decrease in PW1 expression in isolated islet cells led to an increased number of actively cycling beta cells. These observations are consistent with the reported function of PW1 in mesoangioblast and glioma cell proliferation and the presence of a specific recognition sequence for PW1 within the cyclin E promoter [6, 28].

Taken together, these findings show that PW1 controls initiation of the beta cell cycle both in vivo and in vitro under different physiological and experimental conditions. Future studies to investigate the role of PW1 as a negative regulator of the beta cell cycle may reveal it to be a novel therapeutic target for increasing the number of endogenous beta cells.

## Electronic supplementary material

Below is the link to the electronic supplementary material.

ESM Fig. 1 (PDF 2.96 mb)

ESM Fig. 2 (PDF 2078 kb)

ESM Table 1 (PDF 52 kb)

ESM Table 2 (PDF 65 kb)

## Abbreviations

E: Embryonic day
P: Postnatal day
INS: Insulin
GCG: Glucagon
PDL: Partial duct ligation
PDX1: Pancreatic and duodenal homeobox 1
PTF1A: Pancreas-specific transcription factor 1a
Sh: Short hairpin
