# Supplementary material for: The zinc finger transcription factor PW1/PEG3 restrains murine beta cell cycling
Source: Diabetologia. 2016 Apr 29;59:1474–9. doi: 10.1007/s00125-016-3954-z (PMC4901110; doi:10.1007/s00125-016-3954-z)
Supplement: Supplementary file 1 — (PDF 2.96 mb) [file 125_2016_3954_MOESM1_ESM.pdf]

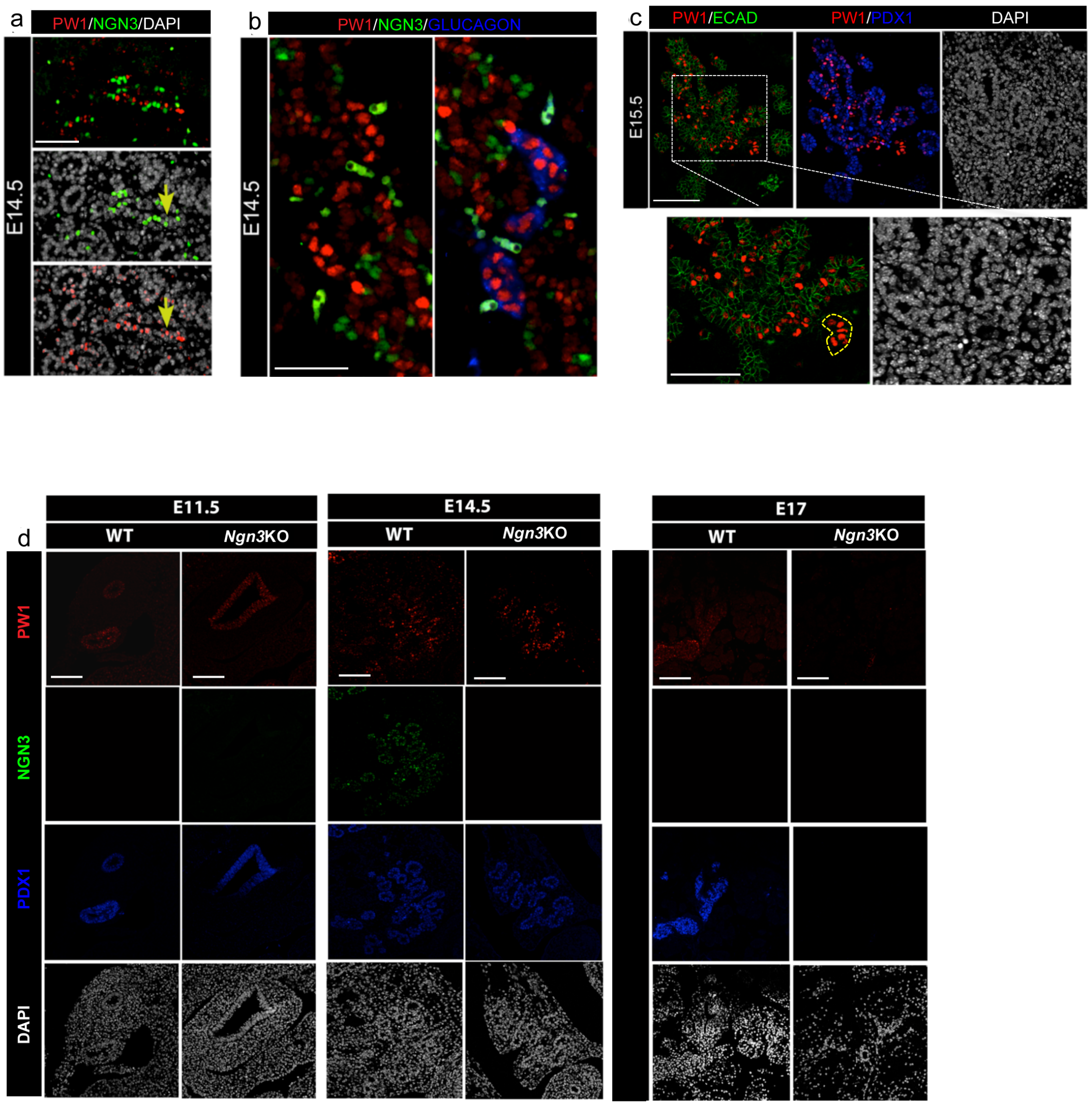

ESM Fig. 1: (a) Expression of PW1 (red) and NGN3 (green) in E14.5 mouse pancreas. Only very rare cells co-express both proteins (yellow arrow). Scale bar: 100µm. (b) Far most NGN3^{+} cells are hormone^{-} and PW1^{-} while most of PW1^{+} cells are hormone^{+} (here GCG in blue). Scale bars: 50µm. (c) At E15.5, E-cadherin expression decreases in PW1^{+} cells (red) near ducts during endocrine cell specification (dashed yellow line). Scale bars: 100µm. (d) PW1 (red) is detected in NGN3 (green) null mouse at E11 and E14.5 but not at E17. Scale bars: 100µm.
