# Supplementary material for: The zinc finger transcription factor PW1/PEG3 restrains murine beta cell cycling
Source: Diabetologia. 2016 Apr 29;59:1474–9. doi: 10.1007/s00125-016-3954-z (PMC4901110; doi:10.1007/s00125-016-3954-z)
Supplement: Supplementary file 2 — (PDF 2078 kb) [file 125_2016_3954_MOESM2_ESM.pdf]

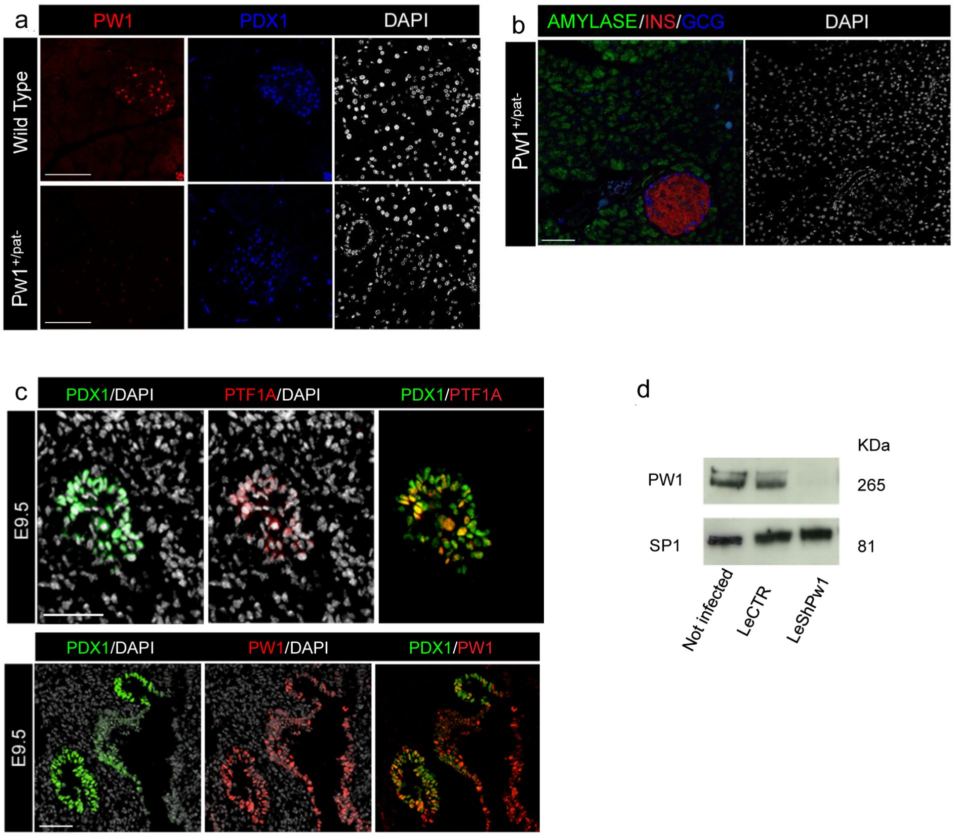

ESM Fig. 2: Expression of PW1 in pancreas of PW1$^{+/pat-}$ and WT mouse. Immunostaining of (a) PW1 (red), PDX1 (blue) and (b) AMYLASE (green), INS (red), GCG (blue) shows normal cytoarchitecture of pancreas from PW1$^{+/pat-}$ 8 weeks old mice. (c) PW1 is expressed in PDX1^{+}PTF1A^{+} cells of E9.5 embryonic pancreas. Nuclear staining: DAPI (white). Scale bars: 100µm. (d) Infection of Min6 cells with LeShPw1 (MOI 100) decreased the level of PW1 protein as compared to control virus transduced cells. SP1, a ubiquitously expressed transcription factor, was used as quality control.
