# Supplementary material for: The zinc finger transcription factor PW1/PEG3 restrains murine beta cell cycling
Source: Diabetologia. 2016 Apr 29;59:1474–9. doi: 10.1007/s00125-016-3954-z (PMC4901110; doi:10.1007/s00125-016-3954-z)
Supplement: Supplementary file 3 — (PDF 52 kb) [file 125_2016_3954_MOESM3_ESM.pdf]

ESM Table1: Primary antibodies

Target	Host	Dilution	Source
CK19	Rat	1:100	DSHB, Iowa City, IA, USA
GFP	Goat	1:200	Abcam, Cambridge, UK
GLUCAGON	Mouse	1:1000	Sigma-Aldrich, St. Louis, MO, USA
INSULIN	Guinea Pig	1:5000	DRC, Brussels, Belgium
KI67	Rat	1:5000	Dako, Glostrup, Denmark
NGN3	Guinea Pig	1:1000	Dr. Gradwohl, INSERM, Strasbourg, France
NKX2.2	Mouse	1:50	DSHB, Iowa City, IA, USA
PAX6	Goat	1:250	Santa Cruz Biotechnology, Heidelberg, Germany
PDX1	Mouse	1:1000	Dr. Wright, Vanderbilt, Nashville, TN, USA
PW1	Rabbit	1:2000	Dr. Sassoon, University of Pierre and Marie Curie, Paris, France
