# Supplementary material for: The zinc finger transcription factor PW1/PEG3 restrains murine beta cell cycling
Source: Diabetologia. 2016 Apr 29;59:1474–9. doi: 10.1007/s00125-016-3954-z (PMC4901110; doi:10.1007/s00125-016-3954-z)
Supplement: Supplementary file 4 — (PDF 65 kb) [file 125_2016_3954_MOESM4_ESM.pdf]

ESM Table 2: PrimeTime qPCR Probes (Integrated DNA Technologies, Coralville, IA USA).

Gene	Probe sequence 5'–3'	Accession number
Cyclin E1	CGCACCACTGATAACCTGAGACCTT	NM_007633.2
Cyclophylin A	TGAGCACTGGAGAGAAAGGATTTGGC	NM_008907
Glucagon	GAGGAACCGGAACAACATfTGCCAAA	NM_008100.2
Insulin1	CATCAGCAAGCAGGTCATTGTTTCA	NM_008386.2
Ki67	CAATCATCAAGGAACGGCCCCAGTC	NM_001081117.2
MafA	CGCACCCAGCCCGGGCACAGGCGGC	NM_194350.1
Neurogenin3	AGAAGCCGGCGCAAGAAGGCCAATG	NM_009719.4
Pdx1	GTGGGCAGGAGGTGCTTACACAGCG	NM_008814.2
Pw1	TTGTGTCATGTGAGAGTGTCG	NM_008817
